# Alteration in glycolytic/cholesterogenic gene expression is associated with bladder cancer prognosis and immune cell infiltration

**DOI:** 10.1186/s12885-021-09064-0

**Published:** 2022-01-03

**Authors:** Yuying Zhang, Baoyi Zhu, Yi Cai, Sihua Zhu, Hongjun Zhao, Xiaoling Ying, Chonghe Jiang, Jianwen Zeng

**Affiliations:** 1 Department of Urology, The Sixth Affiliated Hospital of Guangzhou Medical University, Qingyuan People’s Hospital, Qingyuan, 511518 China; 2 Department of Obstetrics, Shenzhen Longhua Maternity and Child Healthcare Hospital, Shenzhen, 510089 China; 3 Department of Urology, National Clinical Research Center for Geriatric Disorders, Xiangya Hospital, Central South University, No.87 Xiangya Road, Changsha, 410008 China; 4 Department of Translational Medicine, The First Affiliated Hospital of Sun Yat-Sen University, Guangzhou, 51000 China

**Keywords:** Bladder cancer, Glycolysis, Cholesterol synthesis, Metabolic subtypes, Immune microenvironment

## Abstract

**Background:**

Oncogenic metabolic reprogramming contributes to tumor growth and immune evasion. The intertumoral metabolic heterogeneity and interaction of distinct metabolic pathways may determine patient outcomes. In this study, we aim to determine the clinical and immunological significance of metabolic subtypes according to the expression levels of genes related to glycolysis and cholesterol-synthesis in bladder cancer (BCa).

**Methods:**

Based on the median expression levels of glycolytic and cholesterogenic genes, patients were stratified into 4 subtypes (mixed, cholesterogenic, glycolytic, and quiescent) in an integrated cohort including TCGA, GSE13507, and IMvigor210. Clinical, genomic, transcriptomic, and tumor microenvironment characteristics were compared between the 4 subtypes.

**Results:**

The 4 metabolic subtypes exhibited distinct clinical, molecular, and genomic patterns. Compared to quiescent subtype, mixed subtype was more likely to be basal tumors and was significantly associated with poorer prognosis even after controlling for age, gender, histological grade, clinical stage, and molecular phenotypes. Additionally, mixed tumors harbored a higher frequency of *RB1* and *LRP1B* copy number deletion compared to quiescent tumors (25.7% vs. 12.7 and 27.9% vs. 10.2%, respectively, both adjusted *P* value< 0.05). Furthermore, aberrant PIK3CA expression level was significantly correlated with those of glycolytic and cholesterogenic genes. The quiescent subtype was associated with lower stemness indices and lower signature scores for gene sets involved in genomic instability, including DNA replication, DNA damage repair, mismatch repair, and homologous recombination genes. Moreover, quiescent tumors exhibited lower expression levels of pyruvate dehydrogenase kinases 1-3 (PDK1-3) than the other subtypes. In addition, distinct immune cell infiltration patterns were observed across the 4 metabolic subtypes, with greater infiltration of M0/M2 macrophages observed in glycolytic and mixed subtypes. However, no significant difference in immunotherapy response was observed across the 4 metabolic subtypes.

**Conclusion:**

This study proposed a new metabolic subtyping method for BCa based on genes involved in glycolysis and cholesterol synthesis pathways. Our findings may provide novel insight for the development of personalized subtype-specific treatment strategies targeting metabolic vulnerabilities.

**Supplementary Information:**

The online version contains supplementary material available at 10.1186/s12885-021-09064-0.

## Introduction

Bladder cancer (BCa) is one of the most common tumors and the thirteenth leading cause of cancer-related deaths worldwide [1]. Globally, approximately 573,000 patients were diagnosed with BCa in 2020, with 212,000 BCa-related deaths in that year, posing an enormous threat to human health [2]. In recent times, BCa treatment has been revolutionized by emerging therapies such as immunotherapy and molecular-targeted therapy; however, the 5-year survival rate for muscle-invasive BCa remains unsatisfactory [3, 4]. Therefore, continuous understanding of tumor subtyping is desirable to improve the prognostic stratification of BCa to achieve personalized treatment.

Oncogenic metabolic reprogramming is a major hallmark of cancers and allows cancer cells to survive and thrive in harsh conditions [5, 6]. Tumor cells, by an effect known as the Warburg effect, can shift glucose metabolism toward aerobic glycolysis, providing cancer cells with energy and biosynthetic raw materials to promote tumor growth, invasion, and metastasis [5–8]. Metabolic reprogramming also plays a pivotal role in maintaining genomic instability and stemness in cancer cells to allow for self-expansion and resistance to chemotherapy. In addition, glycolytic reprogramming modifies the tumor microenvironment (TME) into a hypoxic, acidic, and nutritionally deficient environment that facilitates cancer cell growth and inhibits immune cell function [5–8]. Therefore, targeting metabolic vulnerability is a promising strategy for cancer therapy.

Pyruvate is the terminal product of glycolysis and servers as a precursor for different biosynthetic pathways. Mitochondrial pyruvate complex (MPC) comprised of pyruvate carriers 1 and 2 (MPC1/MPC2) is the entry point for pyruvate to the mitochondrial matrix for oxidative metabolism, and MPC deficiency contributes to tumor initiation and progression by enhancing glycolysis [9, 10]. In addition to MPC, the pyruvate dehydrogenase complex (PDC) also serves as a gatekeeper in maintaining the balance between anaerobic and aerobic glucose metabolism by catalyzing the conversion of pyruvate to acetyl-CoA for entry into the tricarboxylic acid cycle (TAC) [11]. PDC activity is tightly regulated by pyruvate dehydrogenase kinases (PDKs, isoform 1-4), which can phosphorylate and inactivate the PDC, resulting in attenuated pyruvate oxidative metabolism and increased glycolysis [12]. PDK inhibition has been reported to suppress tumor growth and is a promising therapeutic target for several diseases, including cancers [13, 14].

Recent studies have revealed the remarkable cancer prognosis-determining potential of metabolic heterogeneity [7]. For instance, using glycolytic and cholesterogenic genes, Karasinska et al. classified pancreatic cancer into 4 distinct metabolic phenotypes, which were related to patient survival, and established molecular subtypes [15]. BCa is a heterogeneous malignancy [16], and it is unclear whether it can be stratified into different subtypes through heterogeneity in distinct metabolic pathways to enhance personalized therapy.

In this study, we stratified BCa into 4 distinct metabolic subtypes based on the expression levels of glycolytic and cholesterogenic genes. We aim to clarify the prognostic value of heterogeneity in glycolysis and cholesterol synthesis, determine its association with genomic instability, stemness, and the immune microenvironment in BCa, and provide a research basis for further personalized treatment.

## Materials and methods

### Study datasets and participants

The Cancer Genome Atlas (TCGA) datasets, including RNA-seq expression, single nucleotide variants (SNV) /indels, copy number variation (CNV), and corresponding clinical profiles were downloaded from the GDC portal (https://portal.gdc.cancer.gov/). The GSE13507 microarray dataset was downloaded from the GEO portal (https://www.ncbi.nlm.nih.gov/geo/). The IMvigor210 study, which evaluated the efficacy and safety of PD-L1 inhibitors in locally advanced or advanced urothelial cancer [17, 18], was obtained from http://research-pub.gene.com/IMvigor210CoreBiologies/. A total of 760 primary BCa samples with survival data (TCGA, *n* = 400; GSE13507, *n* = 165; IMvigor210, *n* = 195) were used for this study. The gene expression values for RNA-seq were transformed into TPM (transcripts per kilobase million). RNA-seq and microarray gene expression data were log2 transformed for analysis. Batch effects caused by non-biological technical biases were corrected using the “ComBat” algorithm of “sva” R package. Principal component analysis (PCA) was used to evaluate the batch effect between samples before and after correction. As shown in Supplementary Fig. S1, the PCA confirmed a reduction in batch effects after normalization. The detailed clinical-pathological features of the 3 datasets are shown in Supplementary Table S1.

### Metabolic subtyping

To stratify BCa based on the relative expression levels of genes involved in glycolysis and cholesterol biosynthesis, genes belonging to Reactome gene sets, ‘glycolysis’ (*n* = 72), and ‘cholesterol biosynthesis’ (*n* = 25), were extracted from ‘MsigDB’ (supplementary Table S2). The batch effect-corrected expression values for these genes were standardized by Z-score, and then subjected to consensus clustering (parameters: rps = 1000, pItem = 0.8, pFeature = 1, clusterAlg = hc, distance = euclidean) using the ‘ConsensusClusterPlus’ R package to identify co-expressed glycolysis and cholesterol synthesis genes. The number of clusters was determined according to the criteria of consensus Cumulative Distribution Function (CDF) and the relative change in area under the CDF curve. Samples were divided into 4 metabolic subtypes based on the median expression values of co-expressed glycolytic and cholesterogenic genes i.e., quiescent (glycolytic ≤0, cholesterogenic ≤0); glycolytic (glycolytic > 0, cholesterogenic ≤0); cholesterogenic (glycolytic ≤0, cholesterogenic > 0); mixed (glycolytic > 0, cholesterogenic > 0) subtypes.

### Molecular phenotype classification

Bladder cancer samples were classified into five molecular phenotypes (basal/, luminal, luminal-infiltrated, luminal-infiltration, and neuronal) using the TCGA molecular classifier [19], or into three molecular phenotypes (basal, luminal, and P53-like) using the MD Anderson molecular classifier [20], according to the “BLCAsubtype” R package.

### Tumor microenvironment immune cell infiltration

The CIBERSORT algorithm was used to estimate the relative abundance of 22 tumor-infiltrating immune cell types with the LM22 reference gene signature and 1000 permutations based on TCGA RNA-seq data [21]. The Kruskal-Wallis test was used to compare the degree of immune cell infiltration between the different metabolic subtypes.

### Single-sample gene-set enrichment analysis and stemness index

We performed single-sample gene-set enrichment analysis (ssGSEA) on TCGA samples to estimate the enrichment scores of gene sets involved in maintaining genomic instability [17]. The gene sets included (1) ‘DNA replication,’ (2) ‘mismatch repair,’ (3) ‘base excision repair,’ (4) ‘nucleotide excision repair,’ (5) ‘DNA damage repair,’ (6) ‘homologous recombination,’ and (7) ‘cell cycle’ genes. The stemness index, which was estimated using RNA expression (all available gene sets), was obtained from the UCSC Xena Pan-Cancer Atlas Hub (https://xenabrowser.net/). ANOVA was performed to compare differences in these signature scores between the 4 metabolic subtypes.

### PDKs analysis

Pearson correlation analysis was performed to identify genes significantly correlated with PDK1-3 expression. Genes with |R| > 0.3 and *P* value< 0.001 were considered to be significant. Then, genes significantly correlated with all PDK1, PDK2, and PDK3 were subjected to Gene Ontology (GO) and Kyoto Encyclopedia of Genes and Genomes (KEGG) pathway enrichment analysis using the “clusterProfiler” R package.

### Statistical analysis

R (version 4.0.2) was used for statistical analyses. Kaplan-Meier plots with log-rank test were used to test differences in overall survival using the ‘survival’ and ‘survminer’ tools in the R software package. Cox regression was used to evaluate differences in overall survival after adjusting for potential confounders such as age, gender, histological grade, and cancer stage. The Kruskal-Wallis test, Chi-square test, ANOVA, or Fisher exact test was used for between-group comparisons where appropriate. Values of *P* < 0.05 were considered statistically significant.

## Results

### Distinction of the 4 bladder cancer metabolic subtypes based on the expression levels of glycolysis and cholesterol synthesis genes

**Fig. 1:**
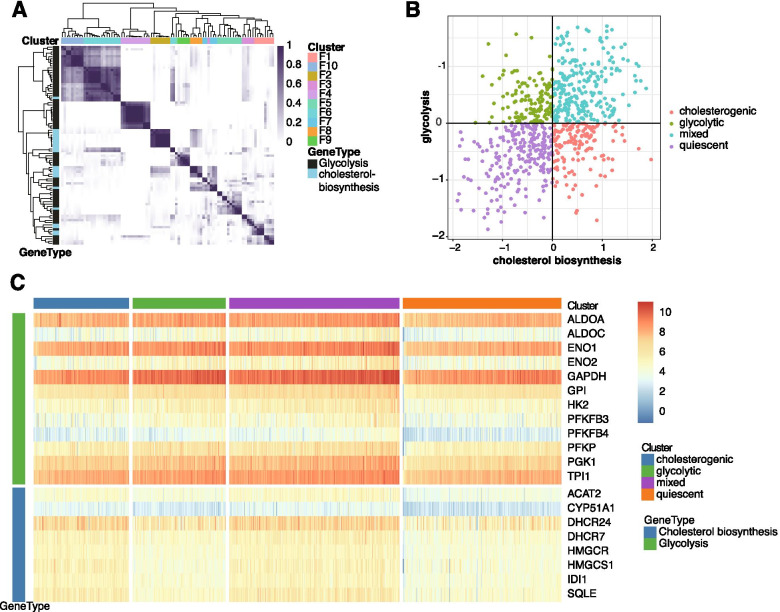
Identification of 4 distinct metabolic subtypes based on expression levels of glycolytic and cholesterogenic genes in BCa. (**A**) Consensus clustering (k = 10) for glycolytic and cholesterogenic genes; (**B**) Scatter plots depicting metabolic subtype proportions based on the median expression levels of glycolytic and cholesterogenic genes; (**C**) Heatmap comparing the expression levels of co-expressed glycolytic and cholesterogenic genes across the 4 subtypes.

As shown in Fig. 1A, ten clusters of co-expressed glycolysis- and cholesterol-related genes were obtained by consensus clustering based on the consensus CDF and change in area under the CDF curve. We identified 2 groups of robustly co-expressed glycolysis (*n* = 12, F4 cluster) and cholesterol synthesis (*n* = 8, F2 cluster) genes which were used for metabolic subtyping (Fig. 1A, Supplementary Table S3). Patients were assigned one of the 4 metabolic subtypes, i.e., quiescent, glycolytic, cholesterogenic, and mixed (Fig. 1B), based on the median expression levels of glycolytic and cholesterogenic genes. The largest proportion of samples exhibited the quiescent subtype (31.1%), followed by the mixed (24.3%), cholesterogenic (22.5%), and glycolytic (22.2%) subtypes. The expression levels of these co-expressed glycolytic and cholesterogenic genes were shown in Fig. 1C.

### Clinical significances of the 4 metabolic subtypes

**Fig. 2:**
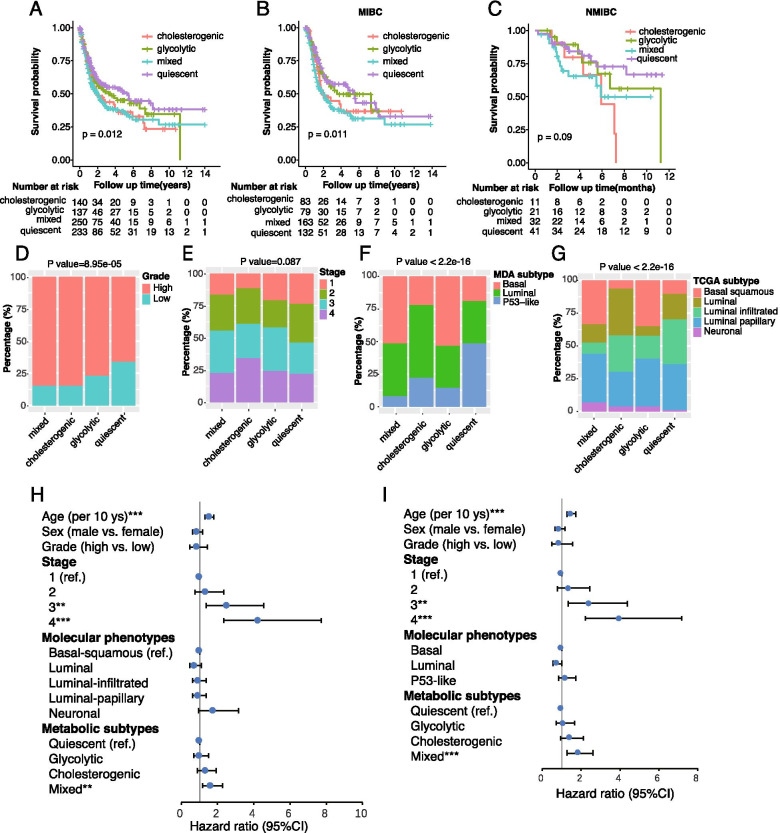
Clinical significances of metabolic subtypes. (**A**-**C**) Kaplan-Meier curves with log-rank test showing the overall survival of patients with (**A**) bladder cancer (*n* = 760), (**B**) muscle-invasive BCa (*n* = 457), and (**C**) non-muscle-invasive BCa (*n* = 105), stratified by metabolic subtypes; (**D**-**E**) Distribution of patients according to (**D**) histological grade, (**E**) tumor stage, (**F**) MDA molecular phenotypes, and (**G**) TCGA molecular phenotypes stratified by 4 metabolic subtype;. (**H**-**I**) Forest plot depicting the result of multivariate cox-regression model. The IMvigor210 dataset was not used in the cox-regression analysis due to missing information on stage and grade. **P < 0.01, ***P < 0.001

As shown in Fig. 2A, the 4 metabolic subtypes demonstrated significant differences in overall survival (log-rank *P* value = 0.012). The quiescent subtype exhibited the best survival, while the mixed exhibited shorter survival. The survival benefit of quiescent tumors in comparison to mixed tumors was observed in muscle-invasive BCa (overall log-rank *P* = 0.011); however, the difference was marginally significant in non-muscle-invasive BCa (log-rank *P* = 0.09), probably due to the limited sample size. (Fig. 2B-C). In addition, significant differences in the histological grade were observed across the 4 metabolic subtypes (*P* = 8.95e-5) and quiescent tumors tended to exhibit lower histological grade and less advanced pathological stage, although the latter did not reach statistical significance (*P* = 0.087) (Fig. 2D-E). Moreover, significant differences in molecular phenotypes were also observed across the 4 metabolic subtypes (*P* < 2.2e-16). Using the MDA molecular classifier, basal tumors were more common in the mixed and glycolytic subtypes (Fig. 2F). Similar findings were observed using the TCGA molecular classifier. Luminal (luminal infiltrated, luminal papillary, and luminal) tumors were more common in the quiescent and cholesterogenic subtypes, while basal-squamous tumors were more common in the mixed and glycolytic subtypes (Fig. 2G). Furthermore, multivariable cox regression revealed that mixed tumors remained an independent predictor for poorer prognosis after controlling for age, gender, histological grade, clinical stage, and molecular phenotypes (Fig. 2H-I and supplementary Table S4). These data indicate that tumors with higher rates of glycolysis and cholesterol synthesis may be more aggressive than tumors with a quiescent subtype, and the metabolic subtype based on glycolytic and cholesterogenic genes may be a promising classifier for prognostic stratification of BCa.

### Genetic alterations of glycolytic and cholesterogenic genes across the 4 metabolic subtypes

**Fig. 3:**
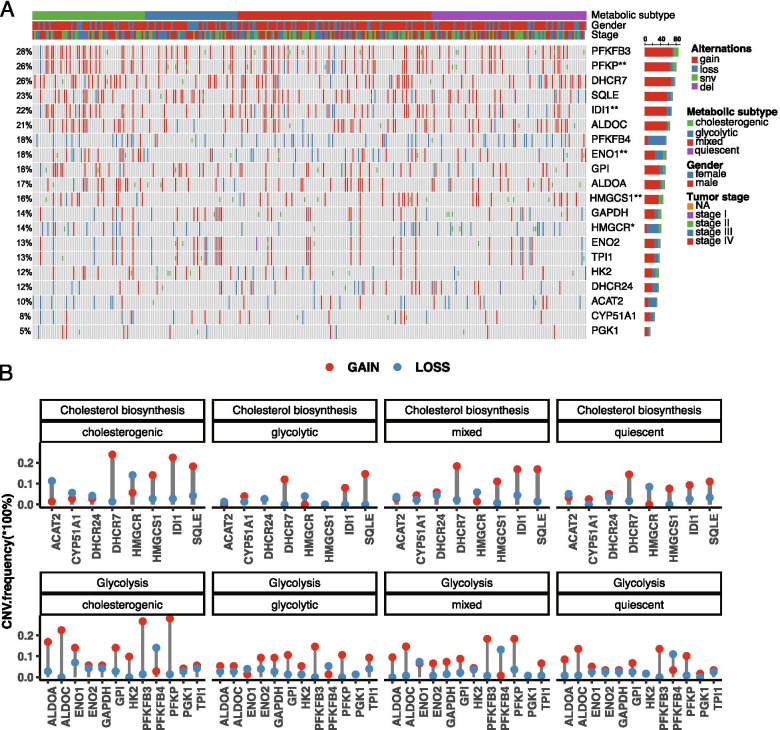
Mutational and CNV profiles of co-expressed glycolysis and cholesterogenic genes across bladder cancer metabolic subtypes in the TCGA study. (**A**) Oncoprint depicting the distribution of SNV/indel and CNV events in the co-expressed glycolytic and cholesterogenic genes across the 4 metabolic subtypes. Fisher exact test was performed to compare the frequencies of alteration across 4 subtypes. *P < 0.05; **P < 0.01; (**B**) CNV variation frequency of the co-expressed glycolytic and cholesterogenic genes in the TCGA cohort. Column height represents the alteration frequency, the red dot indicates the amplification frequency, and the blue dot indicates the deletion frequency

To determine the genetic alteration events associated with metabolic subtypes, we investigated the frequency of SNVs, indels, and CNVs of the 20 co-expressed glycolytic and cholesterogenic genes in the TCGA cohort. As shown in Fig. 3A, CNVs commonly occurred while SNV and indel mutations were rare. The SNV and indel mutation frequencies of the genes were comparable across the four metabolic subtypes (Supplementary Table S5). Of note, CNV gain was more frequently observed than CNV loss across the 4 metabolic subtypes (Fig. 3B), consistent with the upregulated expression levels of these genes. Significant differences in frequencies of CNV alterations were observed in glycolytic genes *ENO1* (*p* = 0.009), *PFKP* (*P* = 0.004), and cholesterogenic genes *HMGCR* (*P* = 0.016), *HMGCS1* (*P* = 0.003), and *IDI1* (*P* = 0.008) across the 4 metabolic subtypes (Fig. 3A and Supplementary Table S6), indicating that aberrant expression of these genes may be related to the development of BCa.

### Association between metabolic subtypes and tumor genomic alterations

**Fig. 4:**
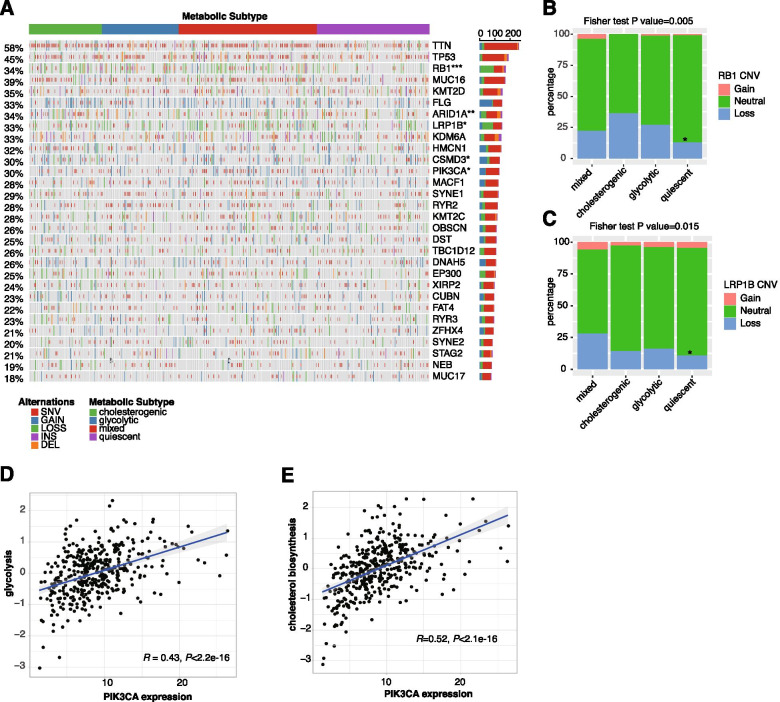
Mutational and CNV profiles of the 30 most frequently altered genes across the 4 metabolic subtypes of bladder cancer in the TCGA study. (**A**) Oncoprint illustrating the distribution of SNV/indel and CNV events affecting frequently altered genes in BCa across the 4 metabolic subtypes. Fisher exact test was performed to compare the frequencies of alteration across 4 subtypes. *P < 0.05; **P < 0.01;***P < 0.001; (**B**-**C**) Distribution of CNV alterations in *RB1* and *LRP1B*. The Fisher exact test was used for comparison. * *P* < 0.05 compared to mixed subtype; (**D**-**E**) Scatter plot showing the correlation between *PIK3CA* expression and the median expression levels of the glycolytic and cholesterogenic genes

Genomic alterations are capable of driving tumor metabolic reprogramming [22, 23]. For instance, oncogenic *PIK3CA* mutations have been reported to reprogram metabolism in a variety of cancers including BCa [23, 24]. In this study, we investigated the genomic alteration (SNV, indel, and CNV) landscapes associated with metabolic subtypes in the TCGA dataset. Of the 30 most frequently altered genes in BCa, the highest frequencies of alteration (SNVs, indels, and CNVs) were observed in *TTN* (58%), *TP53* (45%), and *MUC16* (39.0%). Significant differences in the frequencies of alteration in *RB1* (*P* < 0.001), *ARID1A* (*P* = 0.008), *LRP1B* (*P* = 0.018), *CSMD3* (*P* = 0.011), and *PIK3CA* (*P* = 0.018) were observed across the 4 metabolic subtypes (Fig. 4A, Supplementary Table S7). Of note, CNV loss was frequently observed in *RB1* and *LRP1B*, both of which exhibited the lowest frequencies in the quiescent subtype (Fig. 4B-C). Furthermore, correlation analysis revealed a significant correlation between the expression levels of these genes and the median expression of glycolytic and cholesterogenic genes (Supplementary Table S8). Of these genes, *PIK3CA* expression exhibited the strongest correlation with the expression of glycolytic and cholesterogenic genes (R = 0.43 and 0.52, respectively, and both *P* values were < 0.001) (Fig. 4D-E). These findings are compatible with the notion that *PIK3CA* drives tumor metabolic reprogramming and promotes tumor progression [23]. In summary, this study revealed a distinct genomic alteration landscape across the 4 metabolic subtypes, suggesting the importance of crosstalk between genome instability and metabolic reprogramming in the development of BCa.

### Association between genomic instability or cancer stemness index and metabolic subtypes

**Fig. 5:**
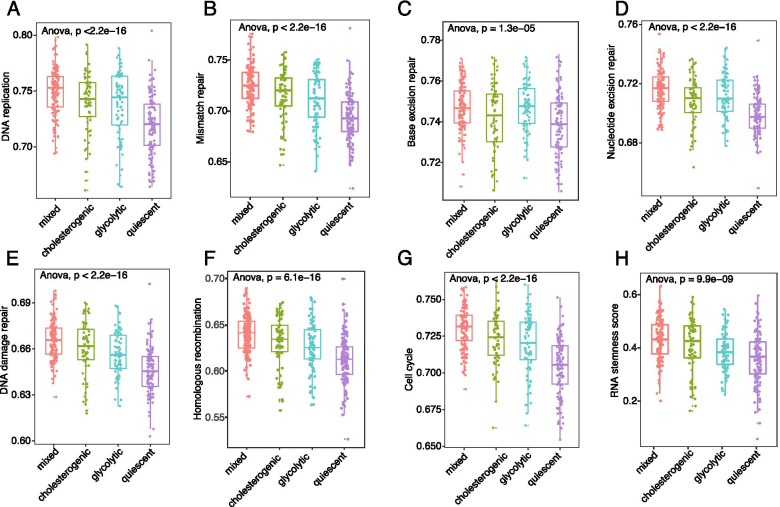
Metabolic subtypes associated with genomic instability and stemness index. (**A-H**) Box and dot plot illustrating the distribution of signature scores of (**A**) DNA replication, (**B**) mismatch repair, (**C**) base excision repair, (**D**) nucleotide excision repair, (**E**) DNA damage repair, (**F**) homologous recombination, (**G**) cell cycle, and (**H**) stemness index across the 4 metabolic subtypes

To further investigate the relationship between genome instability and metabolic subtypes, the signature scores of gene sets involved in genomic instability, including DNA replication, mismatch repair, base excision repair, nuclear excision repair, DNA damage repair, homolog recombination, and cell cycle genes, were compared. As shown in Fig. 5A-G, significant differences were observed between the 4 metabolic subtypes, with the lowest scores generally observed in the quiescent and mixed subtypes, indicating a close relationship of glycolysis and cholesterol biosynthesis with genomic instability that drives tumorigenesis and therapeutic resistance. Furthermore, using the mRNA-based stemness indices derived from TCGA RNA-seq data, we observed the lowest and highest stemness indices in the quiescent and mixed subtypes, respectively (Fig. 5H). In summary, these data indicate a close relationship between metabolic reprogramming and genomic instability and identify glycolysis and cholesterol synthesis as potential targets for controlling BCa.

### TME infiltrating cells and metabolic subtypes

**Fig. 6:**
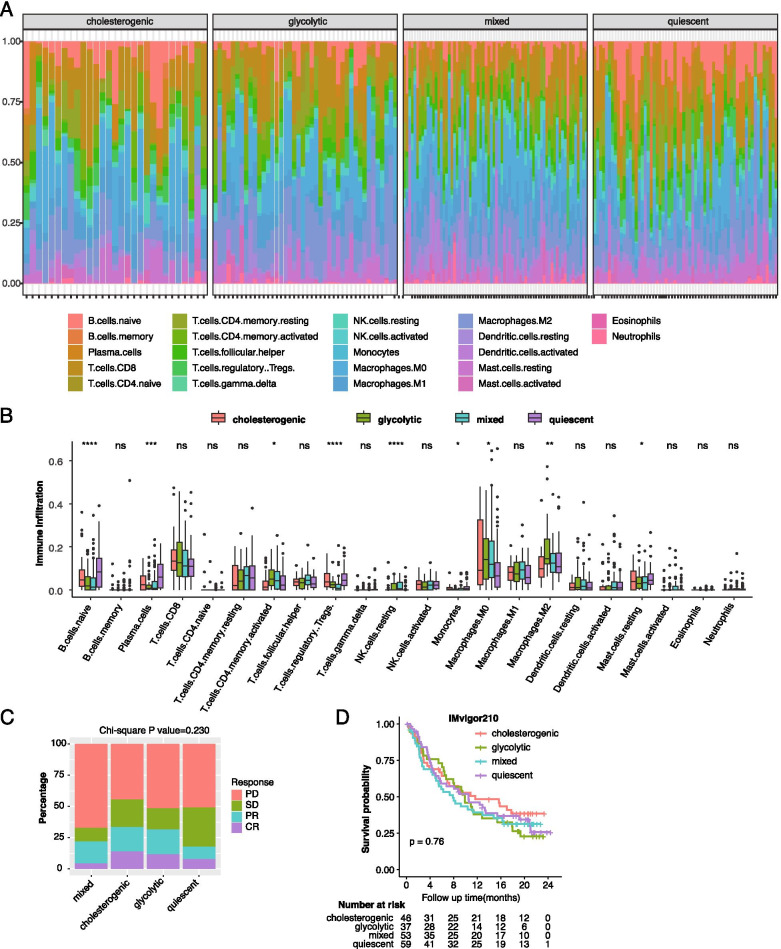
Immune cell infiltration and immunotherapy response across the bladder cancer metabolic subtypes. (**A**-**B**) Bar plot and histogram illustrating the distribution of 22 immune cell types estimated by CIBERSORT across the 4 metabolic subtypes. The Kruskal-Wallis test was used for comparison; (**C**) Immune-response rate according to metabolic subtypes in the IMvigor210 cohort; (**D**) Kaplan-Meier curves with log-rank test showing the overall survival of patients who received PD-L1 immunotherapy stratified by metabolic subtypes in the IMvigor210 cohort

Infiltrating immune cells are an important component of the TME and play a critical role in carcinogenesis and tumor therapeutic response [25]. In this study, using the CIBERSORT algorithm, distinct infiltration patterns were observed across the 4 metabolic subtypes in the TCGA dataset (Fig. 6A-B). Among the 22 immune cell types, naive B cells, plasma cells, CD4 memory-activated T cells, regulatory T cells (Tregs), resting NK cells, monocytes, macrophages (M0/M2), and resting mast cells demonstrated significant differences in infiltrating abundances across the 4 subtypes. Of note, the quiescent and cholesterogenic subtypes exhibited lower M0/M2 macrophage infiltration than the glycolytic and mixed subtypes, and high M0/M2 macrophage levels were associated with poor prognosis (Supplementary Fig. S2). These data indicate that while both glycolysis and cholesterol synthesis contribute to shaping the TME to facilitate tumor growth, glycolysis may have a more significant effect than cholesterol synthesis. We also investigated the association between metabolic subtype and immunotherapeutic response in the IMvigor210 study. However, no significant differences in response rate and survival benefit were observed between the 4 metabolic subtypes (Fig. 6C-D). Further studies with larger sample sizes are needed to confirm these findings.

### Association between MPC1/2 or PDK1-4 alteration and metabolic subtypes

**Fig. 7:**
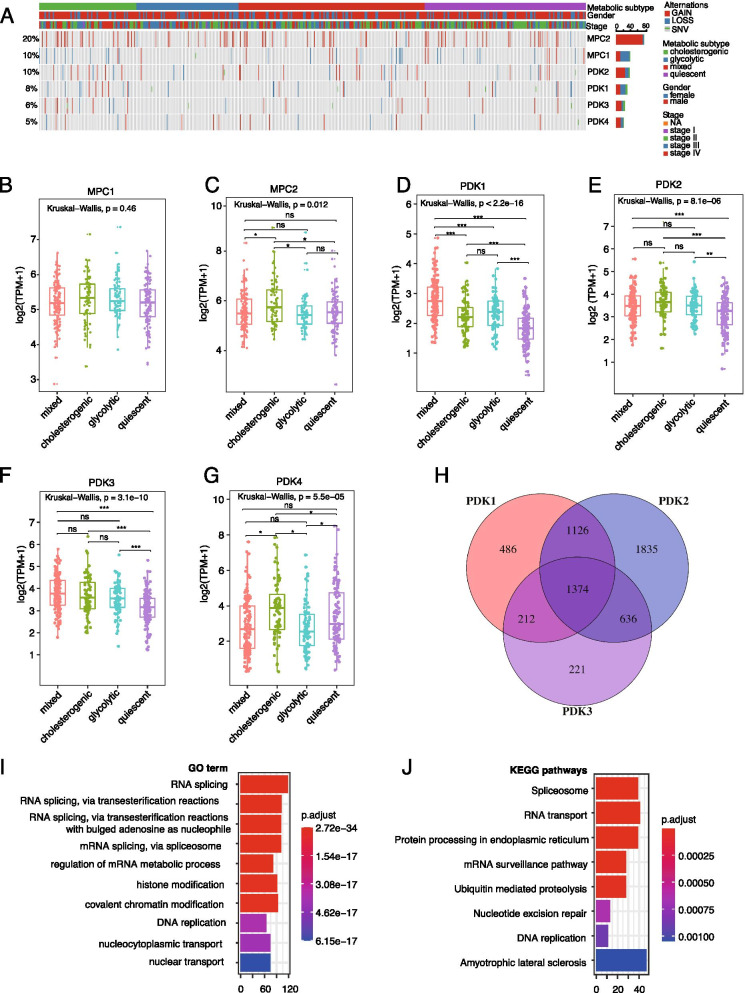
Association between *MPC1*/2 or *PDK1-4* and bladder cancer metabolic subtypes. (**A**) The distribution of *MPC1*, *MPC2,* and *PDK1-4* mutation and CNVs across the 4 metabolic subtypes in the TCGA study; (**B-G**) Expression levels of MPC1, MPC2, PDK1, PDK2, PDK3, and PDK4 across the 4 metabolic subtypes in TCGA cohort; (**H**) Veen diagram showing the number of genes significantly correlated with PDK1, PDK2, or PDK3 in the TCGA cohort (Pearson correlation |R| > 0.3 and *P* value< 0.001). All the common genes (*n* = 1374) were positively associated with PDK1, PDK2, and PDK3; (**I**-**J**) Top 10 most significantly enriched gene sets of GO terms and KEGG pathways associated with PDK1, PDK2, and PDK3 expression. Only genes significantly correlated with PDK1, PDK2, and PDK3 (*n* = 1374) were subjected to enrichment analysis.

Because pyruvate is the terminal product of anaerobic glycolysis and acts as a precursor for different biosynthetic pathways, including cholesterol biosynthesis, we further investigated MPC and PDKs, both are critical players involved in pyruvate processing. The mitochondrial pyruvate carrier (MPC) complex, which consists of MPC1 and MPC2, is required for efficient glucose production, and decreased MPC activity in tumors enhances glycolytic activity, resulting in tumor progression [10]. In TCGA BCa samples, *MPC1* and *MPC2* mutations were rare, with only 1 mutation affecting *MPC1* observed in a glycolytic subtype patient. In contrast, CNVs were common, with the majority of CNVs being deletions in *MPC1* and amplifications in *MPC2*, although no significant differences in *MPC1/2* CNV frequencies were observed between the 4 metabolic subtypes (Fig. 7A). Likewise, no significant differences in the expression levels of MPC1 were observed between the subtypes; however, the expression levels of MPC2 were lower in the glycolytic and mixed phenotypes as compared to the cholesterogenic subtype (Fig. 7B-C).

Similarly, SNV and indel mutations were rare but CNVs were common in *PDK1-4*; however, no significant differences in rates of genetic alteration (CNV or mutation) were observed between the 4 subtypes (Fig. 7A). Nevertheless, *PDK* gene expression levels were significantly different between the 4 metabolic subtypes. Overall, quiescent subtypes exhibited the lowest PDK1-3 expression levels (Fig. 7D-G). Because PDK1-3 expression levels demonstrated the most significant correlations with metabolic subtypes and were significantly lower in quiescent tumors compared to mixed tumors, we further performed Pearson correlation analysis to identify genes significantly correlated with PDK1, PDK2, and PDK3 expression. As shown in Fig. 7H-J, a total of 1374 shared genes were identified to be significantly correlated with PDK1, PDK2, and PDK3 (all Pearson correlation R > 0.3, and *P* value< 0.001), and these genes were significantly enriched in spliceosome, RNA transport, nucleotide excision repair, DNA replication, RNA splicing, et al., in consistence with our findings that metabolic subtypes were tightly associated with genomic instability. In summary, these data suggest that decreased PDC activity resulting from increased PDK mRNA levels may contribute to the development of the malignant features associated with metabolic subtypes, presenting PDKs as potential targets for controlling BCa through the manipulation of metabolic vulnerability.

## Discussion

In this study, based on the expression levels of genes involved in glycolysis and cholesterol synthesis, we identified 4 metabolic subtypes, which demonstrated distinct clinical and molecular characteristics, and different TME immune cell infiltration patterns, although no significant differences were observed in immunotherapy response rate. In summary, our study unveils the importance of metabolic reprogramming in BCa and presents metabolic heterogeneity-based subtyping as a potential prognosis biomarker for personalized therapy.

Glucose metabolic reprogramming is essential for tumor growth and therapeutic resistance [26]. Studies have shown that glycolysis is closely related to BCa development. For instance, upregulation of pyruvate kinase M2 is closely related to tumor growth and chemo-resistance and serves as a potential tumor marker for BCa monitoring [27, 28]. Lactic acid dehydrogenase (LDHA) is a key enzyme in glycolysis and its upregulation in BCa promotes glycolysis, thereby facilitating tumor growth and immune evasion [29, 30]. Aside from glycolysis, increasing evidence shows that cholesterol metabolites also play critical roles in cancer development [31, 32]. Increased cholesterol biosynthesis is a hallmark of many cancers, promoting cancer cell growth and immune evasion by activating cellular signalings such as sonic hedgehog, Notch and receptor tyrosine kinases, and LXR-α signaling [33]. Based on the expression levels of glycolytic and cholesterogenic genes, Karasinska et al. identified 4 distinct metabolic phenotypes with remarkable prognostic significance [15]. As with previous studies, this study also revealed a close relationship between metabolic subtypes and BCa prognosis. Notably, the quiescent and mixed subtypes were associated with the best and worst outcomes, respectively. These results indicate that glycolysis and cholesterol synthesis might act synergistically to accelerate tumor progression in BCa.

It has been well recognized that the molecular characteristics of BCa are associated with prognosis and therapeutic responses. Compared to luminal tumors, basal tumors demonstrated unfavorable survival and suboptimal therapeutic response [19, 20]. In this study, basal tumors were more common in the mixed subtype using both TCGA molecular classifier and MDA classifier, consistent with the unfavorable survival associated with mixed tumors, suggesting a close relationship between metabolic reprogramming and molecular phenotypes. More importantly, metabolic subtype remained as a significant predictor for overall survival after controlling for major confounders including molecular phenotypes. Taken together, our study highlights the prognostic value of metabolic subtypes in guiding personalized therapy.

Genomic instability resulting from mutations in DNA repair genes is a hallmark of most cancers and plays a central role in tumor initiation and progression [34, 35]. Recent studies have revealed the close relationship between metabolic reprogramming and cancer genomic instability [36]. For instance, glycolysis was found to contribute to DNA metabolism by providing metabolites essential for the biosynthesis of nucleotides. Some glycolytic products (like L- and D-lactate) and key glycolytic enzymes (like PGAM1 and PKM2) are involved in DNA damage repair [37–39]. Furthermore, the importance of cholesterol biosynthesis in maintaining genome instability has also been reported [40, 41]. Previous studies have reported that genetic defects of the nucleotide excision repair pathway, including ERCC1 deficiency, result in the suppression of cholesterol biosynthesis [42]. In line with previous reports, our study also revealed a close relationship between glycolysis and cholesterol biosynthesis and genomic stability. Of the 4 metabolic subtypes, the quiescent subtype exhibited the lowest activities in mismatch repair, base excision repair, nucleotide excision repair, DNA damage repair, and DNA replication, while the mixed subtype exhibited relatively higher activities than the other subtypes. Moreover, we observed significant differences in genomic alteration patterns between the 4 metabolic subgroups. *RB1* is a well-known tumor suppressor gene, and its deletion can enhance glycolytic metabolism and driving tumor progression [43]. A recent study also identified *LRP1B,* which encodes low-density lipoprotein receptor-related protein 1B, as a novel tumor suppressor, and *LRP1B* deletion was associated with chemotherapy resistance in ovarian cancer [44]. In this study, although the quiescent tumors harbored similarly high mutation rates in most of the interested genes, consistent with findings reported in a previous study [15], it is worthy of note that the quiescent subtype exhibited lower frequency of CNV loss in RB1 and LRP1B than mixed subtypes, in line with the survival benefit of the quiescent subtype. In addition, we observed frequent mutations in *PIK3CA*, which were positively associated with glycolysis and cholesterol synthesis. In consonance with our findings, previous studies have reported that *PIK3CA* mutation promotes tumor progression partly by enhancing glycolysis [45]. In summary, our findings suggest a close relationship of glycolysis and cholesterol synthesis with genomic instability and present glycolytic and cholesterogenic metabolism targeting as a potential therapeutic strategy for BCa treatment.

Accumulating evidence revealed that metabolic reprograming can contribute to tumor progression by creating a hypoxic, acidic, and nutritionally deficient TME [5–8]. However, the TME cell-infiltrating characteristics of distinct metabolic subtypes remain unclear. In this study, the 4 metabolic subtypes exhibited a distinct immune cell infiltration pattern, demonstrating that the glycolytic and cholesterogenic reprogramming is critical in shaping different TME landscapes. Of note, the quiescent subtype exhibited significantly lower M0/M2 macrophage levels than the other subtypes. A recent study also demonstrated that macrophage-promoted tumor growth by regulating tumor cell metabolism, in support of our findings [46]. Furthermore, macrophages have been proved to be critical players in driving cancer cell immune evasion and are closely related to poor prognosis [47]. Therefore, a comprehensive assessment of the metabolic patterns may enhance our understanding of TME cell-infiltrating characteristics. In this study, we speculated that metabolic subtype could predict immunotherapeutic response, however, we did not observe significant differences in the response to PD-L1 immunotherapy between the 4 subtypes in IMvigor210 study. Therefore, further studies are warranted to evaluate the roles of glycolysis and cholesterol synthesis in immunoregulation in BCa. Furthermore, recent studies have reported that as with cancer cells, TME immune cells also undergo metabolic reprogramming to facilitate tumor growth and immune evasion [48, 49]. In this study, although we demonstrated a distinct TME cell-infiltration pattern associated with heterogeneity in glycolysis and cholesterol synthesis, we did not investigate the metabolic alteration in TME immune cells, which may also contribute to the alteration in the transcriptome profiles of tumor tissues. Further studies, such as studies with single-cell RNA-sequencing, are needed to investigate the crosstalk between metabolic alterations in tumors and TME cells during tumor initiation and progression.

The MPC deficiency has been linked to tumorigenesis by enhancing glycolysis and may serve as a potential target of anticancer therapy by manipulating glycolytic activity [9, 10]. In line with previous reports on other cancer types [15], this study revealed that in BCa, MPC1 was frequently deleted, while MPC2 was mostly amplified. Although no significant differences in MPC1 expression levels were observed between the 4 metabolic subtypes, the decreased *MPC2* expression in the glycolytic and mixed subtypes suggests that *MPC2* may contribute to mitochondrial pyruvate uptake in BCa. In addition to MPC, the PDC also plays a pivotal role in regulating energy homeostasis [50, 51]. Studies have suggested that metabolic reprogramming in cancer cells is associated with PDC inhibition due to phosphorylation of its E1a subunit by PDKs [14, 52, 53]. Thus, inhibition of PDK has been recognized as an attractive strategy in anticancer therapy [54, 55]. While the roles and mechanisms of PDK1-3 in BCa remain unknown, enhanced PDK4 expression in BCa has been reported in previous studies, and PDK4 inhibitors were found to suppress BCa cell invasiveness and to potentiate cisplatin-induced cell death [56, 57]. In this study, we observed significantly lower PDK1-3 expression levels in the quiescent subtype as compared to the other subtypes, consistent with the notion that PDKs contribute to tumorigenesis by inhibiting PDC activity [54, 55]. Furthermore, GO and KEGG enrichment analysis revealed that PDK1-3 was correlational with the critical biological process involved in tumorigenesis, including nucleotide excision repair, DNA replication, RNA splicing, etc., presenting PDK1-3 as potential therapeutic targets for BCa. However, the decreased expression levels of PDK4 in the mixed and glycolytic subtypes did not match our inferences and was inconsistent with other reports [56, 57]. Further studies are needed to investigate the roles played by PDKs in BCa development.

Our study had some limitations. Firstly, we only addressed the intertumoral glycolytic and cholesterogenic heterogeneity, however, the intratumoral metabolic heterogeneity and the comprehensive landscape of tumor metabolism remained uninvestigated. Secondly, the current study is largely based on correlation analysis and there is a lack of experimental validation; therefore, the findings of this study should be interpreted with caution. Thirdly, although we combined 3 cohorts to obtain a large sample size, the sample size in the subgroup analysis of non-muscle-invasive BCa was still small.

Overall, this study identified a metabolic heterogeneity-based classifier with distinct molecular and immune characteristics, and predicted outcomes in BCa, providing novel insights for the development of personalized therapeutic strategies targeting metabolic vulnerabilities.

## Supplementary Information

**Additional file 1: Table S1.** Clinical characteristics of TCGA, GSE13507, and IMvigor210 datasets included in this study. **Table S2.** List of Reactome glycolysis and cholesterol-biosynthesis gene sets. **Table S3.** List of co-expressed glycolysis and cholesterol biosynthesis genes identified by consensus clustering. **Table S4.** Multivariate Cox regression determining the association between metabolic subtypes and overall survival. **Table S5.** Frequency of SNV and indels mutation of the 20 co-expressed glycolytic and cholesterogenic genes in the TCGA dataset. **Table S6.** Frequency of CNV alterations of the 20 co-expressed glycolytic and cholesterogenic genes in the TCGA dataset. **Table S7.** Frequency of genetic alterations of the 30 most frequently altered genes across the 4 metabolic subtypes of bladder cancer in the TCGA dataset. **Table S8.** Correlations between expression of glycolytic and cholesterogenic genes and the 30 most frequently altered genes in TCGA dataset.

**Additional file 2: Figure S1.** The Principal component analysis of samples of TCGA, GSE13507, and IMvigor210 before and after batch effect correction.

**Additional file 3: Figure S2.** Kaplan-Meier curves showing the overall survival of patients stratified by high- and low levels of M0 or M2 macrophages infiltration.

## Data Availability

The current study analyzed publicly available datasets which can be found in https://xena.ucsc.edu/welcome-to-ucsc-xena (TCGA); https://www.ncbi.nlm.nih.gov/gds/?term=GSE13507 (GSE13507); http://research-pub.gene.com/IMvigor210CoreBiologies/ (IMvigor210).

## Abbreviations

BCa: Bladder cancer
TME: Tumor microenvironment
MPC: Mitochondrial pyruvate complex
PDC: Pyruvate dehydrogenase complex
TAC: Tricarboxylic acid cycle
PDKs: Pyruvate dehydrogenase kinases
ssGSEA: Single-sample gene-set enrichment analysis
PCA: Principal component analysis
GO: Gene Ontology
KEGG: Kyoto Encyclopedia of Genes and Genomes
MDA: MD Anderson
