# Supplementary figures and images for: Alteration in glycolytic/cholesterogenic gene expression is associated with bladder cancer prognosis and immune cell infiltration
Source: BMC Cancer. 2022 Jan 3;22:2. doi: 10.1186/s12885-021-09064-0 (PMC8722165; doi:10.1186/s12885-021-09064-0)

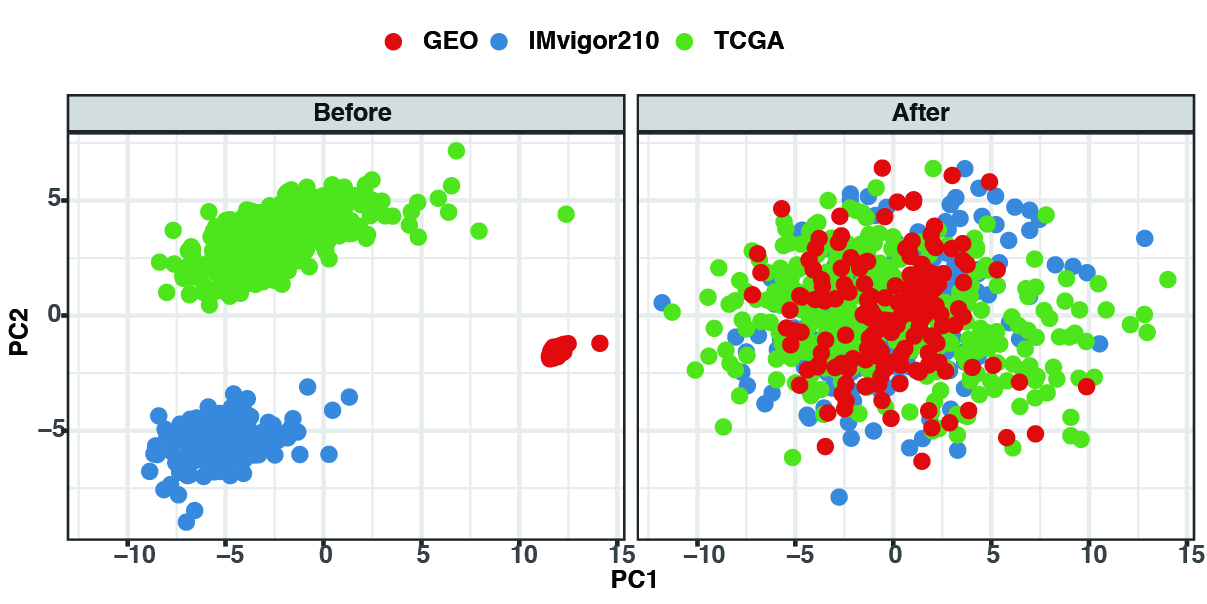

Supplement: Supplementary file 2 — Additional file 2: Figure S1. The Principal component analysis of samples of TCGA, GSE13507, and IMvigor210 before and after batch effect correction. [file 12885_2021_9064_MOESM2_ESM.jpg]

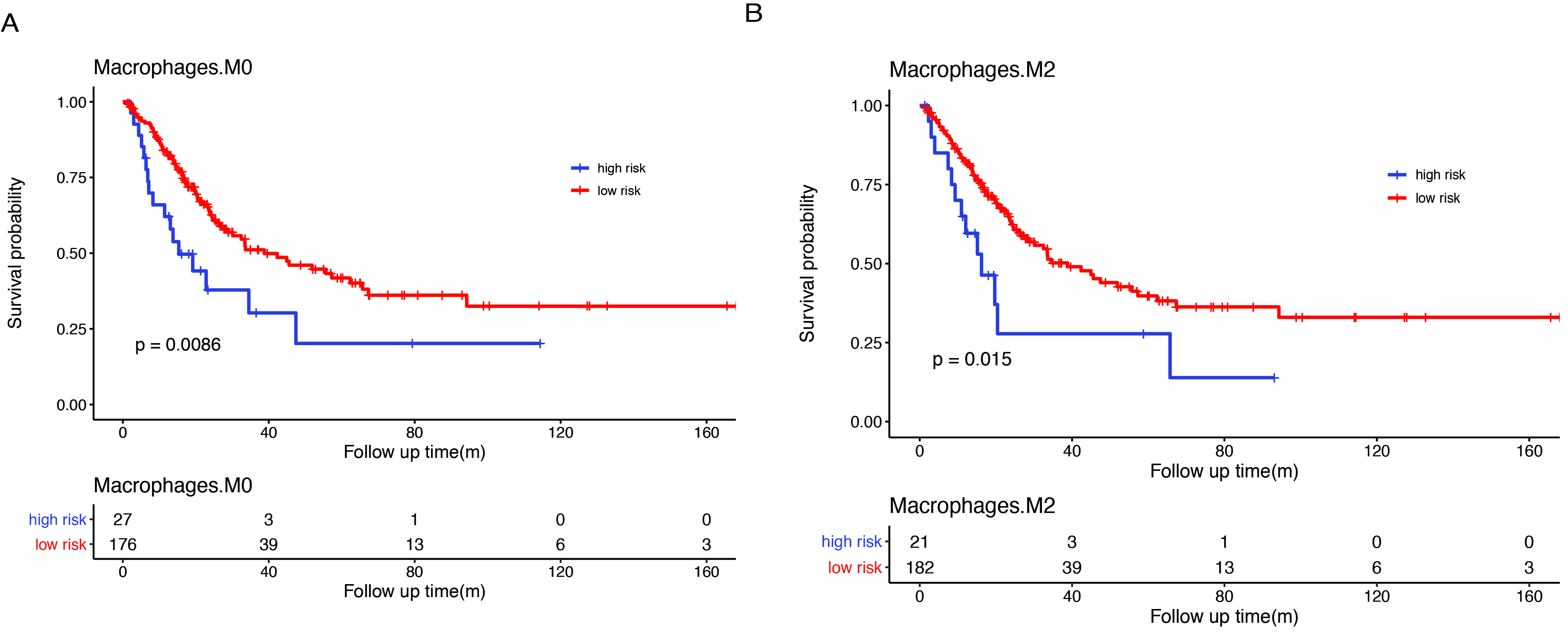

Supplement: Supplementary file 3 — Additional file 3: Figure S2. Kaplan-Meier curves showing the overall survival of patients stratified by high- and low levels of M0 or M2 macrophages infiltration. [file 12885_2021_9064_MOESM3_ESM.jpg]
